# Deep Anomaly Detection Framework Utilizing Federated Learning for Electricity Theft Zero-Day Cyberattacks

**DOI:** 10.3390/s24103236

**Published:** 2024-05-20

**Authors:** Ali Alshehri, Mahmoud M. Badr, Mohamed Baza, Hani Alshahrani

**Affiliations:** 1 Department of Computer Science, University of Tabuk, Tabuk 71491, Saudi Arabia; 2 Department of Network and Computer Security, College of Engineering, SUNY Polytechnic Institute, Utica, NY 13502, USA; badrm@sunypoly.edu; 3 Department of Electrical Engineering, Faculty of Engineering at Shoubra, Benha University, Cairo 11629, Egypt; 4 Department of Computer Science, College of Charleston, Charleston, SC 29424, USA; bazam@cofc.edu; 5 Department of Computer Science, College of Computer Science and Information Systems, Najran University, Najran 61441, Saudi Arabia; hmalshahrani@nu.edu.sa

**Keywords:** smart cities, smart grids, electricity theft, privacy preservation, anomaly detection, zero-day attacks

## Abstract

Smart power grids suffer from electricity theft cyber-attacks, where malicious consumers compromise their smart meters (SMs) to downscale the reported electricity consumption readings. This problem costs electric utility companies worldwide considerable financial burdens and threatens power grid stability. Therefore, several machine learning (ML)-based solutions have been proposed to detect electricity theft; however, they have limitations. First, most existing works employ supervised learning that requires the availability of labeled datasets of benign and malicious electricity usage samples. Unfortunately, this approach is not practical due to the scarcity of real malicious electricity usage samples. Moreover, training a supervised detector on specific cyberattack scenarios results in a robust detector against those attacks, but it might fail to detect new attack scenarios. Second, although a few works investigated anomaly detectors for electricity theft, none of the existing works addressed consumers’ privacy. To address these limitations, in this paper, we propose a comprehensive federated learning (FL)-based deep anomaly detection framework tailored for practical, reliable, and privacy-preserving energy theft detection. In our proposed framework, consumers train local deep autoencoder-based detectors on their private electricity usage data and only share their trained detectors’ parameters with an EUC aggregation server to iteratively build a global anomaly detector. Our extensive experimental results not only demonstrate the superior performance of our anomaly detector compared to the supervised detectors but also the capability of our proposed FL-based anomaly detector to accurately detect zero-day attacks of electricity theft while preserving consumers’ privacy.

## 1. Introduction

The smart grid signifies a revolutionary advancement in the conventional power grid structure, thus incorporating sophisticated digital technology to improve efficiency, dependability, and sustainability [1]. The smart grid facilitates bidirectional communication between utility providers and consumers through real-time two-way communication and data analytics, thereby cultivating a more responsive and adaptable energy environment [2,3]. This modernization enhances the monitoring, control, and optimization of electricity distribution, thus decreasing energy wastage and enhancing overall grid performance [4]. Furthermore, smart grids support the assimilation of new sources like solar and wind power, thereby contributing to sustainable energy resources [5,6]. The smart grid’s rapid fault detection and response capabilities enhance resilience, thus minimizing downtime during power outages and elevating overall system reliability. Consumers also gain increased control over their energy consumption through smart meters (SMs) and immediate feedback, thus encouraging energy conservation and cost-effectiveness. Essentially, the smart grid marks a crucial stride toward a more intelligent, efficient, and environmentally aware energy infrastructure [7].

However, in smart grids, some malicious consumers can manipulate their SMs to report lower metering readings to the electric utility company (EUC) with the aim to decrease their consumption bills [8,9,10,11,12,13,14,15]. Unfortunately, such electricity theft causes high financial losses and overloads the power grids, thus negatively impacting power grid stability all over the world [16]. The electricity theft issue presents a significant global challenge for EUCs, thus resulting in an annual loss that exceeds 96 billion USD due to Non-Technical Losses (NTLs), as emphasized in the study by Wen et al. [17]. For instance, In Canada, the yearly economic losses stemming from energy theft were found to be approximately 100 million USD [18], and in the United States, the figure rose to 6 billion USD [17]. The losses associated with energy theft not only disrupt energy generation but also distribution. Given the adverse impacts of energy theft, the smart grid community has had a consistent and heightened concern regarding the need for effective and comprehensive energy theft detection [19,20].

In the context of a smart grid, energy consumption data could be collected from consumers, and the identification of energy theft can be accomplished through the implementation of conventional machine learning (ML) models, as well as more sophisticated deep learning (DL) models [21]. While these models demonstrate effective performance in detection, a prevailing practice involves transmitting and storing energy consumption data to a cloud data center for the training and execution of detection models. This widely adopted approach raises significant apprehensions regarding the security and privacy of the data [22,23]. Addressing this challenge, *federated learning (FL)* emerges as a novel ML/DL approach that enables collaborative learning from extensively distributed confidential data without the need for data to leave clients’ devices [24,25,26]. In addition to preserving data privacy, FL has the added benefit of allowing access to diverse data types, thereby enhancing the overall performance of models [27,28,29]. Few works in the literature introduced FL-based electricity theft detection frameworks. However, these works suffer from several limitations, as we will discuss in Section 5.

In this paper, to address the existing works’ limitations, we propose a comprehensive FL-based deep anomaly detection framework tailored for practical, reliable, and privacy-preserving energy theft detection. We design our electricity theft detector as an anomaly detector, i.e., it is trained only on benign electricity consumption readings aiming at detecting any deviations from the learned pattern as forms of electricity theft cyberattacks for the following reasons. The first is practicality: due to the absence/limited availability of real malicious electricity consumption readings, it is more practical to design an anomaly detector than to design a supervised one by assuming some cyberattacks to simulate the electricity theft scenarios. The second is robustness: training a supervised detector on specific cyberattack scenarios results in a robust detector against those attacks but it might fail to detect new attack scenarios. Therefore, it is critical to design an anomaly detector to detect not only known attacks but also zero-day attacks.

Although a few works in the literature investigated anomaly detectors for electricity theft, none of the existing works address the consumers’ privacy. To the best of our knowledge, this work is the first to investigate an FL-based deep anomaly detection framework for electricity theft zero-day cyberattacks. Our proposed framework enables EUCs to detect electricity theft zero-day attacks while maintaining the consumers’ privacy. The main contributions in this paper are summarized as follows:

- We investigate the performance of various DL-supervised electricity theft detectors, including the feedforward fully connected neural network (FCNN), convolutional neural network (CNN), and long short-term memory (LSTM) recurrent neural network (RNN), against electricity theft zero-day attacks. Our experimental results demonstrate that while supervised detectors are successful against known attacks, they fail to detect novel unknown attacks.
- We investigate the performance of various anomaly detection models, including Isolation Forest (IF), One-Class Support Vector Machine (OCSVM), and Autoencoder, against electricity theft zero-day attacks. Our experimental results indicate that the autoencoder-based detector outperforms the IF- and OCSVM-based detectors. Moreover, our results demonstrate a significant improvement in the anomaly detectors’ performance compared to the supervised detectors in defending against electricity theft zero-day attacks.
- We propose a comprehensive FL-based deep anomaly detection framework tailored for practical, reliable, and privacy-preserving energy theft detection. In our framework, an EUC aggregation server initializes the parameters of a fully connected feedforward autoencoder (FC-AE)-based anomaly detector and distributes it to selected consumers to participate in FL. In each FL round, participating consumers train copies of the anomaly detector locally based on their historical electricity consumption readings and only share their local detectors’ parameters with the aggregation server to update the global detector by averaging the local detectors’ parameters. Our experimental results demonstrate the capability of our proposed FL-based anomaly detector to accurately detect unknown (zero-day) attacks of electricity theft while protecting consumers’ privacy.

The rest of this article is organized as follows. In Section 2, we elaborate on the system and threat models considered in this paper. Section 3 discusses our proposed FL-based anomaly detection framework of electricity theft cyberattacks. Section 4 presents the results of the experiments and discusses them. Section 5 discusses the related works and their limitations. Finally, Section 6 concludes this article.

## 2. System and Threat Models

This section provides the system model and the adversary attacks.

### 2.1. System Model

**Figure 1:**
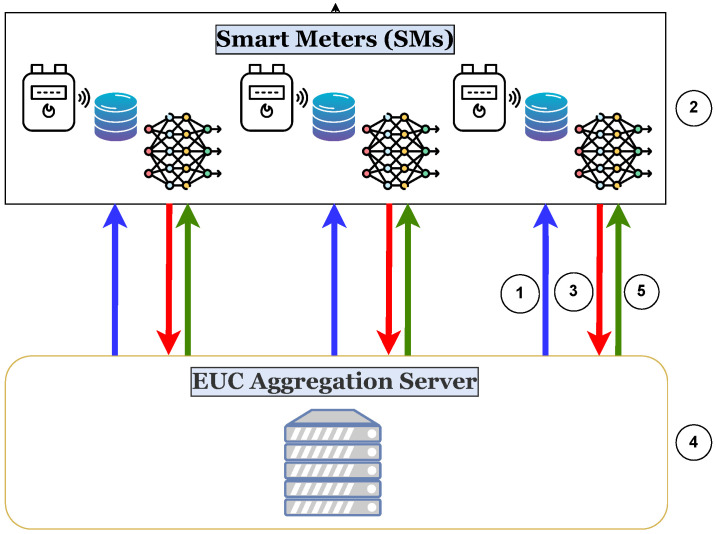
An illustration of our FL-based deep anomaly detection framework. (1) Initial global model distribution. (2) Local model training. (3) Local model parameters upload. (4) Local models’ parameters aggregation. (5) Distribution of the updated global model.

Figure 1 illustrates the system model considered in this paper, which comprises two main entities: consumers with SMs installed at their premises and an aggregation server at the EUC. Consumers’ SMs can record fine-grained electricity usage readings, such as one reading every 30 min, which could potentially leak sensitive information if revealed. Therefore, consumers may be unwilling to share their fine-grained consumption readings with the EUC due to privacy concerns. On the other hand, the EUC aims to mitigate electricity theft cyberattacks through an anomaly detector that requires thorough training on historical electricity consumption readings. To address this dilemma of training a global anomaly detector while preserving consumers’ privacy, the EUC does not request consumers to share their private data directly, but instead, they participate in an FL process. The interactions between the EUC server and the participating consumers in the FL process are depicted in the following figure and summarized through the following steps:

- *Step 1*: The EUC aggregation server initializes the global anomaly detector parameters and distributes them to the participating consumers.
- *Step 2*: The consumers train the copies of the global detector locally using their private and sensitive data.
- *Step 3*: After training, the consumers upload their local detectors to the EUC aggregation server for aggregation.
- *Step 4*: The aggregation server averages the received local detectors’ parameters to update the global detector parameters.
- *Step 5*: The new global detector is distributed back to the consumers for the next FL training iteration.

The above explanation implies the practicality of implementing our proposed framework in terms of training data requirements, infrastructure requirements, computational costs, communication costs, and scalability considerations:

- *Training Data Requirements*: Due to privacy concerns, it is more practical for EUCs to use FL than to ask consumers to share their private data. Due to the unavailability of real malicious data and the requirement to detect zero-day attacks, it is more practical for EUCs to train anomaly detectors than supervised detectors trained only on specific attack scenarios.
- *Infrastructure Requirements*: Our framework’s infrastructure requirements are based on the current smart grids’ advanced metering infrastructure (AMI) comprising SMs installed at consumers’ premises, data centers, and communication networks connecting the consumers’ side to the EUC side. Thus, our proposed framework is practical, as it does not require EUCs to implement costly and drastic changes to their infrastructure.
- *Computational Costs*: The computational costs of our framework are very reasonable. On the consumers’ side, all that is required from a participating consumer in the FL process is to train a local model on their small private data. This cheap process takes a few minutes on a device with modest computational resources. On the EUC side, all that is required is to implement an aggregation process to update the parameters of the global detector. This is a very simple task given the EUC’s high computational capabilities.
- *Communication Costs*: Our framework is communication-efficient. Unlike traditional methods that require consumers to upload their private data to the EUC, thus consuming AMI communication resources, our framework only requires consumers to upload their trained model parameters.
- *Scalability Considerations*: Considering factors such as performance, flexibility, cost, and complexity, our framework demonstrates excellent scalability. The number of consumers will not adversely affect any of these factors. Our framework does not necessitate all smart grid consumers to participate in the FL process or is designed to function only if a specific number of consumers is met. Particularly, consumers may be clustered into representative groups based on factors including geographical location, home size, number of inhabitants, and cost of living, and a few consumers from each group are selected to participate in the FL process [12,15].

### 2.2. Adversary Model

**Figure 2:**
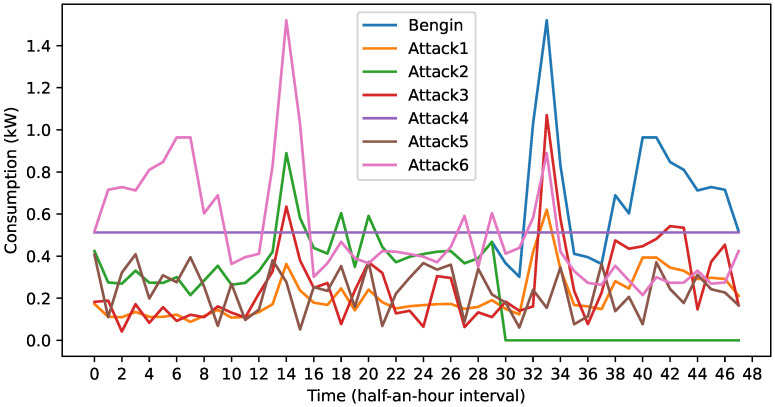
Visualization of a benign electricity usage sample and the corresponding malicious samples.

We assume that malicious consumers attempt to compromise their SMs to report reduced electricity readings to minimize their electricity bills. However, unlike the majority of the existing works that assume specific cyberattacks to be launched by malicious consumers to commit electricity theft, in our work, we devise an anomaly detection framework to detect any form of electricity theft. To validate our work, we assume that attackers may launch a group of cyberattacks, including but not limited to the six attacks common in the literature [30]. The attacks considered in this work are visualized in Figure 2 and given in Table 1. In such attacks, $Rep_{c}\left(d,t\right)$ and $Rec_{c}\left(d,t\right)$ denote the reported and recorded electricity readings, respectively, by a consumer *c*’s SM during a specific time interval *t* on a given day *d*.

Attack 1 describes a form of persistent attack, where a malicious consumer always multiplies the real electricity consumption reading by a reduction factor $r_{1}$ before reporting it, where $r_{1}$ is a random number greater than zero and less than one. On the contrary, attack 2 describes a form of a discontinuous attack, where a malicious consumer reports zero electricity consumption readings in a randomly selected interval between $t_{s}\left(d\right)$ and $t_{e}\left(d\right)$ while reporting the real readings outside that interval. Attack 3 describes another form of a persistent attack, where a malicious consumer always reports scaled-down versions of the real electricity consumption readings by a time-dependent factor $r_{2}$ that is greater than zero and less than one. Apart from the previous attacks, attacks 4, 5, and 6 describe a set of attacks exploiting the fact that dynamic electricity pricing is employed in smart grids, where the electricity tariff is varied over time. In attack 4, a malicious consumer always reports a constant reading throughout the day, which is the expected mean of the electricity consumption readings throughout the day, where $(Rec_{c}\left(d\right)$ represents a vector of electricity consumption readings of a day *d*. Instead of reporting fixed readings, in attack 5, the attacker launches a more stealthy attack by reporting scaled versions of $mean\left(M_{c}\left(d\right)\right)$ by a time-dependent factor $r_{3}$ that is greater than zero and less than one. Finally, in attack 6, a malicious consumer commits electricity theft by reporting low electricity consumption readings when the electricity prices are high and high electricity consumption readings when the prices are low.

## 3. Proposed Framework

This section illustrates the underlying rationale behind the development of our innovative FL-based deep anomaly detection framework, which has been specifically customized for the robust, trustworthy, and privacy-conscious detection of electricity theft. We delve into the framework’s key components and their significance in ensuring practical applicability, reliability, and privacy preservation.

### 3.1. Rationale behind the Design

*Anomaly Detector vs. Supervised Detector:* We designed our electricity theft detector as an anomaly detector, i.e., its training solely focuses on benign electricity consumption data with the goal of detecting deviations from the learned pattern as forms of electricity theft cyberattacks for the following reasons. The first is practicality: because of the absence/scarcity of real malicious electricity consumption readings, it is more practical to design an anomaly detector than to design a supervised one by assuming some cyberattacks to simulate the electricity theft scenarios. The second is robustness: training a supervised detector on specific cyberattack scenarios results in a robust detector against those attacks, but it might fail to detect new attack scenarios. Therefore, it is critical to design an anomaly detector to detect not only known attacks but also zero-day attacks.

*Autoencoders vs. Shallow Models:* Autoencoders are artificial neural networks that can be used in various applications, including feature extraction/dimensionality reduction, new data generation, and anomaly detection [31]. Autoencoders have been chosen to design our anomaly detection system due to several considerations. First, they are unsupervised machine learning techniques, meaning they do not require a labeled dataset to be trained, which makes them well suited to the electricity theft problem, where only benign data are available. Moreover, unlike shallow models, such as one-class support vector machines (OCSVMs), the deep learning (DL) architectures of autoencoders enable them to uncover the underlying electricity consumption patterns and detect the temporal correlations among the electricity consumption readings, which in turn fosters electricity theft detection.

*Federated Learning (FL) vs. Centralized Learning (CL):* Unlike most existing works that adopt CL for training a global electricity theft detector, we adopt FL. In CL, consumers share their historical fine-grained electricity consumption readings with a data aggregation server owned by the electric utility company (EUC) to train a global model on the whole collected data. However, this approach incurs a privacy issue and communication overhead, both of which can be avoided by adopting FL. In FL, consumers do not share their historical electricity consumption readings; instead, they train local models on them while solely sharing the trained models’ parameters with the EUC.

### 3.2. Autoencoders

#### 3.2.1. An Overview

A simple autoencoder generally comprises two elements, one encoder and one decoder, of similar but reciprocal neural network architectures. The purpose of the encoder is to transform an input space into a latent feature space, while the decoder’s purpose is to transform the latent feature space into an output space. In particular, the encoder takes a high-dimensional input sample and produces its compressed representation in the latent space, thereby capturing the essential features. On the other hand, the decoder receives this latent space representation from the encoder and converts it into an output sample matching the dimensionality of the original input. The decoder’s output sample represents a reconstructed form of the encoder’s original input sample, and the autoencoder should be trained to minimize the reconstruction error, which measures the disparity between the reconstructed sample and the original sample.

To develop an autoencoder-based anomaly detector for electricity theft, the autoencoder undergoes two phases. During the training phase, the autoencoder is exclusively trained on benign samples representing genuine electricity consumption readings. In the inference phase, the autoencoder is tested against either benign or malicious samples. The detection of electricity theft is accomplished by comparing the autoencoder’s reconstruction error to a predefined threshold. Since the autoencoder was trained solely on benign samples, testing it against benign samples yields reconstruction errors within a specific range. Conversely, when tested against malicious samples, the reconstruction errors fall outside that range. The threshold is utilized to ascertain whether the reconstruction error of the tested sample falls within the benign range; otherwise, an alarm is triggered for electricity theft.

#### 3.2.2. Fully Connected Feedforward Autoencoder (FC-AE)

**Figure 3:**
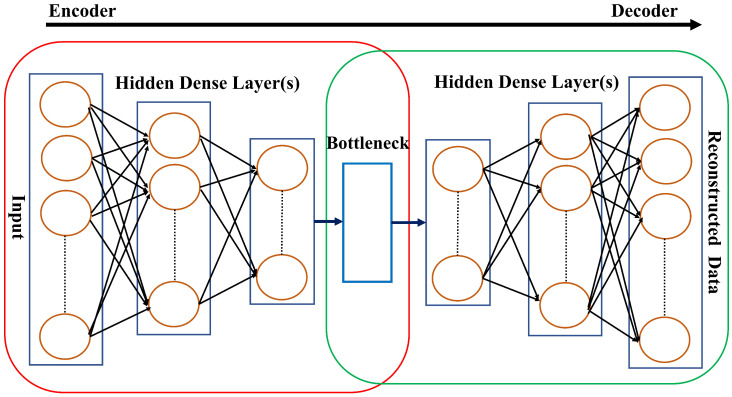
The architecture of a fully connected feedforward autoencoder (FC-AE).

The simplest way to design an autoencoder is to use fully connected dense layers, as shown in Figure 3. In particular, the encoder comprises a stack of fully connected dense hidden layers that process the data received from the input layer towards the bottleneck layer that resembles the latent space representation of the encoder’s input. The bottleneck layer in a standard FC-AE is typically a fully connected dense layer [32,33]. This layer consists of a specific number of neurons representing the dimensionality of the latent space. The decoder comprises a stack of fully connected dense hidden layers that process the data received from the bottleneck layer towards the output layer that resembles the reconstructed version of the encoder’s original input. There are 48 neurons in both the input and output layers resembling the total number of readings in an electricity consumption sample. The sample depicts the daily electricity usage of a consumer, where one reading is logged by the SM every half hour. Let L−2 denote the count of hidden layers in an FC-AE, including the bottleneck layer, and let $N_{l}$ denote the count of neurons in each layer *l*, where l=2,…,L−1, and *L* represent the total count of layers in the autoencoder.

Consider $w_{nn^{\prime }}^{l}$ as the weight associated with the linkage between a neuron *n* within layer *l* and a neuron $n^{\prime }$ within layer l−1 Let $W^{l}$ represent the matrix containing all weights for layer *l*. The term $b_{n}^{l}$ signifies the bias associated with neuron *n* at layer *l*, while $b^{l}$ is a vector encompassing all biases for layer *l*. Consider $o_{n}^{l}$ as the aggregated weighted inputs to neuron *n* within layer *l*, thereby considering the connections $w_{nn^{\prime }}^{l}$ and outputs $a_{n^{\prime }}^{l-1}$ from neurons in layer l−1 along with the bias $b_{n}^{l}$, i.e., $o_{n}^{l}=\sum_{n^{\prime }}w_{nn^{\prime }}^{l}a_{n^{\prime }}^{l-1}+b_{n}^{l}$. The output of neuron *n* at layer *l* is symbolized as $a_{n}^{l}$ and is obtained by applying the activation function σ to $o_{n}^{l}$, which is expressed as $a_{n}^{l}=\sigma \left(o_{n}^{l}\right)$. Using vector notation, the layer *l*’s output is represented as $a^{l}=\sigma \left(o^{l}\right)$, where $o^{l}=W^{l}a^{l-1}+b^{l}$. This signifies that the output $a^{l}$ is obtained by applying the activation function σ to the weighted sum $o^{l}$, where $o^{l}$ is computed as the product of the weight matrix $W^{l}$ and the previous layer’s output $a^{l-1}$ plus the bias vector $b^{l}$. It is important to note that the autoencoder’s output for the input layer is represented as $a^{1}=x$, with *x* being the original input vector to the autoencoder. Additionally, the autoencoder’s output for the output layer is denoted as $a^{L}=x^{\prime }$, where $x^{\prime }$ signifies the autoencoder’s reconstructed output vector based on the input *x*.

Training the autoencoder focuses on finding the best weights and biases for all layers that shrink the difference between *x* and $x^{\prime }$, i.e., the reconstruction error. The autoencoder’s training process can be modeled by the following optimization formula:

(1) $$\min_{\Theta }C=\frac{1}{\left|X_{\mathrm{TR}}\right|}\sum_{i=1}^{|X_{\mathrm{TR}}|}{∥x^{\left(i\right)}-{x^{\prime }}^{\left(i\right)}∥}^{2},$$

where Θ represents the autoencoder’s parameters, including the weights and biases for all layers, *C* is the mean squared error (MSE) loss function used to measure the autoencoder’s reconstruction error, TR denotes the training dataset, and $\left|X_{\mathrm{TR}}\right|$ is the number of training samples. The optimization problem in (1) can be solved using the minibatch gradient descent (MBGD) algorithm. The steps of the MBGD algorithm are repeated for multiple epochs until the convergence. In each epoch, the training dataset TR is divided into minibatches of equal size *B*, where *B* is the number of samples in each batch. In each minibatch *m*, the weights and biases of the autoencoder are updated based on the average gradients of all the samples in the minibatch.

### 3.3. Federated Learning (FL)

Unlike CL, where all training samples are available at an EUC data server to train a global model, in FL, the consumers do not share their private data with the EUC. The FL process is explained in Algorithm 1. The following notations are used in the algorithm. The term $\nabla_{a}C$ signifies the partial derivative of the loss function with respect to *a*. The symbol ⊙ denotes an elementwise product operation. $\sigma^{\prime }\left(o^{l}\right)$ expresses the reciprocal of the partial derivative of $o^{l}$ with respect to $a^{l}$. The notation T indicates a matrix transpose operation. As Algorithm 1 implies, the FL process unfolds in three main stages: initialization, training, and aggregation. In the initialization stage, an EUC aggregation server undertakes the task of initializing the parameters, such as weights and biases, of the global model. It then selects *N* consumers to participate in the FL training and distributes the global model to the selected consumers. Following this distribution, the aggregation server collaborates with the enlisted consumers over multiple FL rounds to iteratively refine the global model until convergence is achieved. Within each round, the consumers engage in the training stage, while the aggregation server oversees the aggregation of model updates.
**Algorithm 1:** Training of the FC-AE using FL.
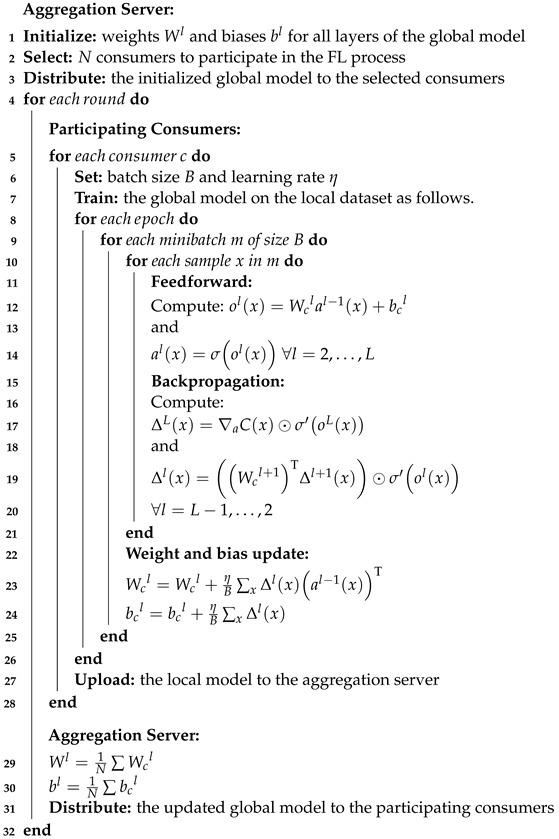


In the FL training stage, each consumer undertakes the task of training the received copy of the global model locally using their private dataset. Typically, this process involves employing the MBGD algorithm, where consumers specify the batch size and learning rate and train the local model across multiple epochs until convergence is attained. Within each epoch, consumers segment their local training dataset into minibatches of uniform size *B*. Within every minibatch *m*, two key steps are performed. In the first step, for every sample *x* in *m*, the sample is propagated forward through the autoencoder layers to calculate the output vector from each layer $a^{l}\left(x\right)$. Subsequently, a backpropagation is initiated to calculate error terms $\Delta^{l}\left(x\right)$ for all layers l=L−1,…,2. In the second step, for every sample *x*, the gradients of the loss function *C* for the weights and the biases are computed as functions of the error terms $\Delta^{l}$ [34]. Following this, the weights and biases of all the autoencoder layers are updated utilizing the average gradients computed for all the samples in *m*. Once completed, consumers upload the parameters of their local models to the aggregation server.

In the aggregation stage, the aggregation server computes the mean of all the participating consumers’ weights and biases to adjust the parameters of the global model, as outlined in Algorithm 1. Following this, the aggregation server distributes the global model to consumers to commence a new iteration of the FL process.

## 4. Performance Evaluation

### 4.1. Dataset

This study makes use of authentic electricity consumption data sourced from the Irish Smart Energy Trial (ISET) dataset [35]. The dataset comprises data gathered from SMs installed in roughly 3600 residential properties. Over a span of 536 days, these SMs recorded electricity usage at 30 min intervals, thus yielding a comprehensive dataset with an average of approximately 25,728 readings per consumer. Some consumers have been randomly selected from the ISET dataset to be considered in our experiments. This wealth of data provides a strong basis for the development and evaluation of our models designed to detect electricity theft. This study is focused on detecting electricity theft daily. Therefore, the readings from the ISET dataset have been utilized to create benign electricity consumption samples of 48 readings each. For the malicious samples, representing electricity theft cases, they were created from the benign samples utilizing the attacks explained in Section 2.2.

### 4.2. Metrics

We employed the following metrics to assess the performance of our trained electricity theft detectors:

- **Accuracy (*ACC*)**: It is the proportion of correctly identified samples among the total classified samples. Its value is derived from the following equation:
  (2) $$ACC\left(\%\right)=\frac{TP+TN}{TP+TN+FP+FN}\times 100,$$
  where TP represents the accurately identified malicious samples, which are denoted as true positives. Similarly, TN signifies the correctly identified benign samples, which are termed as true negatives. Conversely, FP refers to the misidentified benign samples, which are designated as false positives, while FN indicates the misidentified malicious samples, which are termed as false negatives.
- **Precision (*PR*)**: It gives the proportion of true electricity theft samples to the tally of samples identified by the detector as theft. Its value is derived as follows:
  (3) $$PR\left(\%\right)=\frac{TP}{TP+FP}\times 100$$
- **Detection Rate (*DR*)**: It is the proportion of the correctly identified electricity theft samples among all the tested malicious samples. Its value is derived as follows:
  (4) $$DR\left(\%\right)=\frac{TP}{TP+FN}\times 100$$
- **False Alarm (*FA*)**: It is the proportion of misidentified benign samples as electricity theft among all the tested benign samples. Its value is derived as follows:
  (5) $$FA\left(\%\right)=\frac{FP}{FP+TN}\times 100$$
- **Highest Difference (*HD*)**: It is simply the subtraction of FA from DR. Its value is derived as follows:
  (6) HD(%)=DR(%)−FA(%)
- **False Negative Rate (*FNR*)**: It is the proportion of the misidentified electricity theft samples as benign among all the tested malicious samples. Its value is derived as follows:
  (7) $$FNR\left(\%\right)=\frac{FN}{FN+TP}\times 100.$$
- **F1 score (*F*1)**. It represents the harmonic mean of PR and DR. Its value is derived as follows:
  (8) $$F1\left(\%\right)=\frac{2*PR*DR}{PR+DR}\times 100$$
- **Receiver Operating Characteristic (ROC) Curve**: It graphically illustrates the connection between the TP and FP rates across various classification thresholds.
- **Precision–Recall (P–R) Curve**. It graphically illustrates the connection between PR and recall across various classification thresholds.

### 4.3. Experiments

In this paper, we have implemented two experiments. The first experiment evaluated the performance of supervised detectors versus anomaly detectors against electricity theft zero-day attacks. The second experiment evaluated the performance of the FL-based anomaly detector compared to the CL-based anomaly detector.

#### 4.3.1. Experiment 1

In this experiment, the daily electricity consumption samples of all consumers were combined to form the benign dataset. Then, we shuffled the benign samples and divided them into training and testing parts with a ratio of 2 to 1. The training benign samples are considered as the training dataset for anomaly detectors. As the datasets required for training supervised and anomaly detectors are different, we prepared the training dataset for supervised detectors as follows. To begin with, we divided the training benign samples into three parts. We treated attacks 1, 3, and 5 as already known attacks and attacks 2, 4, and 6 as unknown attacks. Then, we used attacks 1, 3, and 5 to generate equal numbers of malicious samples from the three parts of benign training samples. Finally, we combined the benign and malicious samples to create the training dataset for supervised detectors. After that, we trained various existing supervised detectors, including FCNN, CNN, and LSTM, that perform at an almost state-of-the-art level in detecting known electricity theft cyberattacks. Moreover, we trained three anomaly detectors, including IF, OCSVM, and FC-AE. Moving to the evaluation phase, we used the following steps to prepare the testing dataset: (1) dividing the test benign samples into six parts, (2) using attacks 1 through 6 to generate corresponding malicious samples for each part, and (3) combining the benign and malicious samples. Then, we tested the performance of all the trained electricity theft detectors on zero-day attacks. The experimental results are given in Table 2.

Table 2 compares the performance of anomaly detectors to the performance of existing supervised detection models under zero-day attacks using various evaluation metrics: ACC, DR, HD, FNR, and F1. Supervised detectors such as CNN, LSTM, and FCNN are known for their high ability to detect known electricity theft cyberattacks. However, as we can observe from the table, these detectors suffered under zero-day attacks. Considering the CNN detector as an instance, we can notice that it achieved 60.73% for the ACC, 26.83% for the DR, 21.47% for the HD, 40.59% for the F1, and 73.16% for the FNR. The low ACC, DR, HD, and F1 scores and low FNR rates demonstrate the limitations of supervised detectors. DR values of approximately 27%, 28%, and 37% for the CNN, LSTM, and FCNN detectors, respectively, indicate their failure to detect most of the tested malicious samples. In other words, FNR values of approximately 73%, 72%, and 63% for the CNN, LSTM, and FCNN detectors, respectively, indicate that these detectors misidentified the zero-day attack samples as begin instead of detecting them as electricity theft. *Upon comparing the results of the anomaly detectors to the supervised detectors in Table 2, it becomes evident that anomaly detectors are superior in countering zero-day attacks related to electricity theft. The higher ACC, DR, HD, and F1 scores and lower FNR rates demonstrate this.*

**Figure 4:**
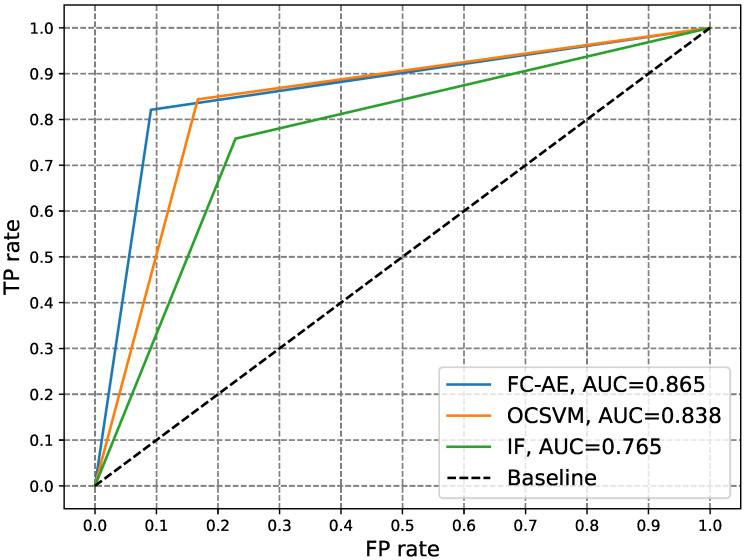
Comparison of the ROC curves of different anomaly electricity theft detectors.

**Figure 5:**
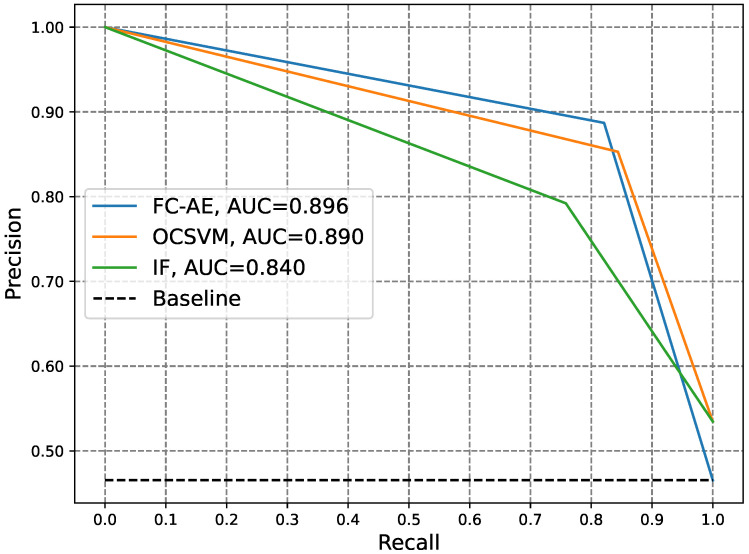
Comparison of the PR curves of different anomaly electricity theft detectors.

Furthermore, to visually compare the three experimented anomaly detectors, IF, OCSVM, and FC-AE, at various classification thresholds, Figure 4 and Figure 5 show the ROC and PR curves of the three detectors, respectively. An analysis of Table 2 along with Figure 4 and Figure 5 reveals that the FC-AE detector emerged as the top-performing anomaly detector among those examined. It demonstrated the highest values for ACC, DR, HD, F1, AUC-ROC, and AUC-P-R while exhibiting the lowest FNR. The superior performance of the FC-AE detector compared to shallow detectors such as IF and OCSVM can be attributed to the DL architecture of autoencoders. This architecture enables the FC-AE detector to unveil intricate electricity consumption patterns within the electricity consumption data, thereby enhancing the detection of electricity theft. Finally, upon comparing the performance of the FC-AE and CNN detectors against zero-day attacks, it becomes evident that the FC-AE anomaly detector achieved superior results. This is evidenced by an approximately 26% increase in the ACC, a 62% increase in the DR, a 53% increase in the HD, and a 45% increase in the F1, alongside a notable 62% reduction in the FNR.

#### 4.3.2. Experiment 2

Unlike Experiment 1, which assumes that consumers share their historical fine-grained electricity consumption readings with the EUC to train a global model, in Experiment 2, they train local models on their private data and only share the parameters of the trained models with the EUC to preserve their privacy. For each participating consumer in the FL, the daily electricity consumption samples have been shuffled and divided into training and test parts with a ratio of 2 to 1. The training benign samples are considered as the training set. Then, to prepare a test set containing zero-day attack samples from each consumer, we followed these steps:

1. Randomly dividing the test benign samples from each consumer into six parts.
2. Using attacks 1 through 6 to generate corresponding malicious samples for each part.
3. Combining the benign and malicious samples.
4. The test samples from all consumers constitute the final test set.

**Figure 6:**
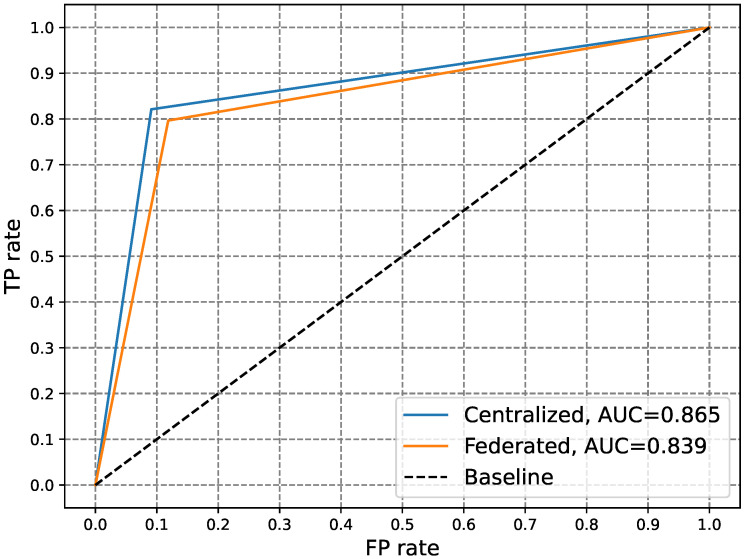
Comparison of the ROC curves of CL- and FL-based anomaly electricity theft detectors.

**Figure 7:**
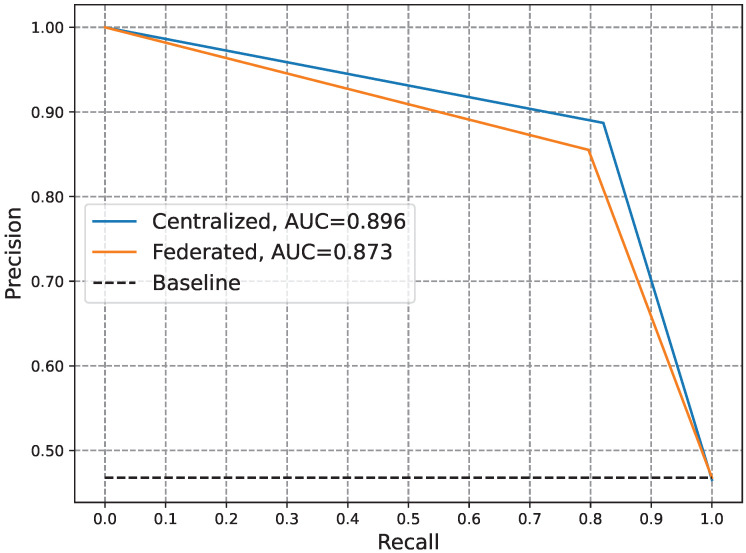
Comparison of the PR curves of CL- and FL-based anomaly electricity theft detectors.

Table 3 compares the performance of the CL and FL-based anomaly detectors in terms of the ACC, PR, DR, FA, HD, FNR, and F1. Moreover, Figure 6 and Figure 7 compare the performance of the two types of detectors in terms of the AUC-ROC and AUC-P-R. The anomaly detector based on CL is deemed optimal when assuming consumers’ willingness to share private data with the EUC. Consequently, the FL-based anomaly detector’s performance should be evaluated in relation to this optimal detector. Analysis of the data and visuals reveals that the FL-based detector achieved comparable performance to its CL-based counterpart. The slight variance in performance between the two detectors is considered an acceptable trade-off for ensuring privacy. This underscores the effectiveness of our FL-based anomaly detector in safeguarding consumer privacy while accurately identifying zero-day attacks related to electricity theft.

## 5. Related Works and Limitations

Several papers in the literature investigated electricity theft detection in smart grids using ML techniques. While most existing works have proposed detecting electricity theft using supervised learning approaches, few works have investigated anomaly detection approaches. In the following subsections, we categorize the existing works into supervised and anomaly detection approaches and review them.

### 5.1. Supervised Detection Approaches

A large number of papers exist under this category; therefore, we survey some prominent examples. In the work presented by [36], a fuzzy SVM was suggested, thus yielding a detection accuracy of only 72%. Another strategy, grounded in supervised learning and detailed in [37], adopted random forests and attained a more favorable detection accuracy of 80%. In contrast, additional methodologies leverage advanced DL techniques, such as RNNs [38] and FCNNs [39], thereby achieving a superior detection accuracy surpassing 90%. Some innovative approaches advocate for a hybrid methodology incorporating RNNs and CNNs. Noteworthy examples of this hybrid approach, as proposed by certain methodologies [40], have demonstrated a detection accuracy within the range of 89%. Despite the effectiveness of ML models in detection, the significance of ensuring the security and privacy of data has escalated. This is particularly crucial, as deployed detection systems are obligated to adhere to diverse regulations and laws, such as the General Data Protection Regulations (GDPR) in Europe [41]. Therefore, few works in the literature introduced FL-based electricity theft detection frameworks [17,42,43,44].

#### FL Works

In a study by Wen et al. [17], the FedDetect framework was introduced. This framework consists of several components, including a data center, a control center, multiple detection stations, and consumers. Within this framework, each detection station is restricted to accessing data solely from local consumers. These employ a local differential privacy mechanism to preprocess their data before transmitting them to their respective detection stations to preserve their privacy. Subsequently, the detection stations collaborate with the control and data centers via FL to collectively train a global model for identifying instances of electricity theft. Moreover, the study outlined in [42] presents a framework resembling FedDetect. However, the authors managed to execute the FL training using a single server, referred to as the aggregation server, in contrast to FedDetect, which employed two servers. Unlike FedDetect, where a temporal convolutional network (TCN)-based detection model was used, the work in [42] used a hybrid model comprising CNN and RNN to enhance the detection performance.

Similar to the work in [42], the research discussed in [43] empowered diverse detection stations to learn from the collective experience of other detection stations facilitated by a central server. Moreover, to enhance the precision with respect to identifying energy theft, the study proposed the utilization of a federated voting classifier (FVC). This classifier employs a majority voting-based consensus derived from conventional ML classifiers, including random forests, bagging classifiers, and k-nearest neighbors. Additionally, the research discussed in [44] introduced a decentralized FL framework specifically tailored for training the detection model used by distribution system operators (DSOs). This framework facilitates the aggregation of model parameters among DSOs without relying on a centralized federated server. The framework also accommodates the possibility of dropout among DSOs during the model training process. Subsequently, within this defined framework, a decentralized federated extreme gradient boosting model was devised specifically for detecting instances of electricity theft.

**Limitations:** The preceding studies face certain limitations. To begin with, they either make assumptions about the existence of trusted electricity theft detection stations while disregarding consumers’ privacy concerns [42,43,44], or they resort to differential privacy techniques to protect consumers’ data from the detection stations [17]; however, this comes with the drawback of sacrificing accuracy in detection. In essence, the studies discussed in [42,43,44] predominantly concentrate on implementing FL among EUCs to develop a global model. They operate under the assumption that each EUC has access to sufficient data on electricity consumption for training a local model. However, they overlook the challenge of how a local model can be trained by an EUC using its consumers’ private data without jeopardizing their privacy [42,44], or they presume that consumers inherently trust their respective EUC [43]. This presumption suggests that consumers prioritize convenience over privacy and are willing to share their private data with the EUC, which may not hold in practice.

Moreover, while the preceding studies focus on training supervised models for detecting electricity theft, a significant challenge in implementing these approaches lies in the need for training datasets encompassing both normal and malicious behaviors. However, the scarcity of authentic malicious datasets specifically tailored for smart grid environments poses a considerable obstacle to the widespread adoption of these techniques. Furthermore, existing research has not addressed the effectiveness of supervised detectors against emerging threats, such as zero-day attacks. Our study addresses this gap by demonstrating that while these detectors perform well against known attacks, they struggle to identify novel attack patterns.

To summarize, although there have been recent examinations into employing FL for identifying electricity theft, the existing frameworks encounter various challenges. Therefore, to advance the current state of the art, we have proposed a comprehensive FL-based anomaly detection framework tailored for practical, reliable, and privacy-preserving energy theft detection. Unlike the above works, our proposed framework is capable of detecting both known and zero-day attacks while preserving the consumers’ privacy by employing anomaly detectors and getting rid of trusted detection stations.

### 5.2. Anomaly Detection Approaches

Anomaly detectors are trained only on benign electricity consumption readings, and they detect any deviations from the learned pattern as forms of electricity theft cyberattacks. A limited number of studies have explored anomaly detection methods for electricity theft [30,32,45,46,47,48]. In [30], Jokar et al. devised an anomaly detection system based on the OCSVM tailored for electricity theft detection. They also devised a multiclass SVM-based detector. Based on their experimental results, they recommend multiclass SVMs over the OCSVM. However, their recommendation is contingent upon the assumption that all methods of electricity theft are already known, which is not practical. Cybersecurity is an ongoing challenge, and attackers can continuously devise new methods to steal electricity. Moreover, Singh et al. [45] investigated the utilization of Principal Component Analysis (PCA) for identifying electricity theft. PCA was employed to reduce the dimensionality of the electricity consumption samples by extracting principle components. Then, in the test phase, an anomaly score was calculated for each sample based on the principle components. Finally, the anomaly scores were compared to a predefined threshold to detect theft. The authors validated their PCA-based approach using attacks 1, 3, and 4. However, they did not test their approach against attacks 2, 5, and 6. Furthermore, Krishna et al. explored the application of an autoregressive integrated moving average (ARIMA) approach for identifying electricity theft as outlined in [46,47]. They proposed checks on both the mean and variance, which effectively reduced the amount of stolen electricity by an attacker by 77.46%.

In contrast to the preceding studies, which rely on statistical techniques such as PCA and ARIMA or shallow ML algorithms like OCSVM, the research outlined in [32,48] employed DL methods. In [32], Takiddin et al. aimed to devise an anomaly detector of electricity theft cyberattacks characterized by an enhanced detection performance compared to the previous anomaly detectors. Therefore, they proposed using autoencoders as anomaly detectors. As we have shown in this paper, autoencoders achieve superior performance compared to shallow detectors such as IF and OCSVM. This is due to the DL architecture of autoencoders that enables them to unveil intricate electricity consumption patterns within the electricity consumption data, thereby enhancing the detection of electricity theft. Furthermore, Habbak et al. introduced the utilization of deep support vector data description (DSVDD) for identifying electricity theft in [48].

**Limitations:** None of the above anomaly detection works address the consumers’ privacy. Therefore, in this paper, we have proposed an FL-based anomaly detection framework to enable EUCs to detect electricity theft zero-day attacks while preserving the consumers’ privacy. None of the existing works also provide a comparative analysis between deep supervised and anomaly detectors against zero-day attacks.

## 6. Conclusions

In this paper, we have proposed an FL-based deep anomaly detection framework tailored for practical, reliable, and privacy-preserving electricity theft detection. In our proposed framework, consumers and an EUC aggregation server collaboratively train an FC-AE-based anomaly detector as follows. The aggregation server initializes the detector parameters and then distributes it to the participating consumers. Consumers train local copies of the detector on their private electricity usage data and only share the trained detectors’ parameters with the server. The server aggregates the received parameters to update the global detector parameters. These steps are repeated until the convergence of the detector is reached. We conducted extensive experiments to prove the merits of our proposed framework. The experimental results indicate the following: (1) While the supervised electricity theft detectors were successful against known attacks, they failed to detect new attacks; (2) Anomaly detectors outperformed supervised detectors in defending against electricity theft zero-day cyberattacks; (3) The capability of our proposed FL-based anomaly detector to preserve consumers’ privacy while accurately detecting zero-day attacks of electricity theft has been demonstrated.

## Figures and Tables

**Table 1:** Electricity theft cyberattacks.

Number	Formulation
Attack 1	${Rep}_{c}\left(d,t\right)=r_{1}Rec_{c}\left(d,t\right)$
Attack 2	${Rep}_{c}\left(d,t\right)=\left\{\begin{array}{cc} 0 & \forall t\in \left[t_{s}\left(d\right),t_{e}\left(d\right)\right]\\ Rec_{c}\left(d,t\right) & \forall t\notin \left[t_{s}\left(d\right),t_{e}\left(d\right)\right] \end{array}\right.$
Attack 3	${Rep}_{c}\left(d,t\right)=r_{2}\left(d,t\right)Rec_{c}\left(d,t\right)$
Attack 4	${Rep}_{c}\left(d,t\right)=mean\left(Rec_{c}\left(d\right)\right)$
Attack 5	${Rep}_{c}\left(d,t\right)=r_{3}\left(d,t\right)mean\left(Rec_{c}\left(d\right)\right)$
Attack 6	${Rep}_{c}\left(d,t\right)=Rec_{c}\left(d,T-t+1\right)$

**Table 2:** Comparison of the performance of supervised and anomaly electricity theft detectors against zero-day attacks.

Approach	Architecture	Metrics				
		ACC	DR	HD	FNR	F1
**Supervised**	**CNN**	60.73	26.83	21.47	73.16	40.59
	**LSTM**	62.55	28.31	25.11	71.68	43.06
	**FCNN**	65.07	36.78	30.14	63.22	51.29
**Anomaly**	**IF**	76.43	75.81	52.96	24.18	77.48
	**OCSVM**	83.87	84.38	67.67	15.61	84.84
	**FC-AE**	86.80	88.70	74.07	11.29	85.28

**Table 3:** Comparison of the performance of CL- and FL-based anomaly electricity theft detectors.

Architecture	Metrics						
	ACC	PR	DR	FA	HD	FNR	F1
**Centralized**	86.80	82.11	88.70	14.62	74.07	11.29	85.28
**Federated**	84.17	80.01	85.51	16.86	68.65	14.48	82.48

## Data Availability

The original contributions presented in the study are included in the article, further inquiries can be directed to the corresponding authors.
